# AKT phosphorylation as a predictive biomarker for PI3K/mTOR dual inhibition-induced proteolytic cleavage of mTOR companion proteins in small cell lung cancer

**DOI:** 10.1186/s13578-022-00862-y

**Published:** 2022-08-02

**Authors:** Ming-Chun Hung, Wan-Ping Wang, Ya-Hui Chi

**Affiliations:** 1 Institute of Biotechnology and Pharmaceutical Research, National Health Research Institutes, 35 Keyan Road, Zhunan, Miaoli County, 35053 Taiwan; 2 Graduate Institute of Biomedical Sciences, China Medical University, 40402 Taichung, Taiwan

**Keywords:** PI3K inhibitor, mTOR, Small cell lung cancer

## Abstract

**Background:**

Constitutive activation of PI3K signaling has been well recognized in a subset of small cell lung cancer (SCLC), the cancer type which has the most aggressive clinical course amongst pulmonary tumors. Whereas cancers that acquire a mutation/copy gain in PIK3CA or loss of PTEN have been implicated in enhanced sensitivity to inhibitors targeting the PI3K/AKT/mTOR pathway, the complexities of the pathway and corresponding feedback loops hamper clear predictions as to the response of tumors presenting these genomic features.

**Methods:**

The correlation between the expression profile of proteins involved in the PI3K/AKT/mTOR signaling and cell viability in response to treatment with small molecule inhibitors targeting isoform-specific PI3Ks, AKT, and mTOR was assessed in 13 SCLC cancer cell lines. Athymic nude mice were used to determine the effect of PI3K/mTOR dual inhibition on the growth of xenograft SCLC tumors in vivo. The activation of caspase signaling and proteolytic cleavages of mTOR companion proteins were assessed using recombinant caspases assays and Western blot analyses.

**Results:**

Our results indicate that the sensitivity of these SCLC cell lines to GSK2126458, a dual PI3K/mTOR inhibitor, is positively correlated with the expression levels of phosphorylated AKT (p-AKT) at Thr308 and Ser473. Inhibition of pan-class I PI3Ks or PI3K/mTOR dual inhibition was shown to induce proteolytic cleavage of RICTOR and RPTOR, which were respectively dependent on Caspase-6 and Caspase-3. A combination of a clinically approved PI3Kα-selective inhibitor and an mTORC1 inhibitor was shown to have synergistic effects in inducing the death of SCLC cells with high p-AKT. We observed no clear correlation between PTEN levels and the survival of SCLCs in response to PI3K/mTOR dual inhibition; however, PTEN depletion was shown to increase the susceptibility of low p-AKT SCLC cells to dual PI3K/mTOR inhibitor-induced cell death as well as the proteolytic cleavage of RICTOR.

**Conclusions:**

These results suggest the level of p-AKT can be a companion diagnostic biomarker for the treatment of SCLC involving the combinational use of clinically approved isoform-specific PI3K and mTOR inhibitors.

**Supplementary Information:**

The online version contains supplementary material available at 10.1186/s13578-022-00862-y.

## Background

Small cell lung cancer (SCLC) accounts for approximately 15% of all lung cancers, resulting in ~ 30,000 deaths each year in the United States. Without treatment, SCLC has the most aggressive clinical course of any type of pulmonary tumor, with median survival from diagnosis of only 2 to 4 months [1]. SCLC patients often present with metastasis at the time of diagnosis, thereby precluding surgery as a treatment option [2]. Since the early 1980s, the standard of care for limited stage (LS-SCLC) and extensive stage (ES-SCLC) disease has involved a drug combination of etoposide with cisplatin or carboplatin [3]. ES-SCLC patients are sensitive to standard chemotherapy and radiation therapy; however, tumors typically recur rapidly after primary treatment, with only 6% of patients surviving 5 years from the time of diagnosis [4]. Since 2019, the FDA has approved chemotherapy in combination with anti-PD-L1 antibodies (atezolizumab or durvalumab) for SCLC. This treatment was shown to provide a slight improvement in median overall survival (12.3 to 13.0 months) compared to chemotherapy alone (10.3 months for ES-SCLC) [5, 6]. Nonetheless, the therapeutic benefits of immune oncology-combined therapy are modest, and no targeted therapy is currently available.

SCLC is most commonly encountered in heavy smokers. The tumor cells express neuroendocrine markers and are believed to derive from cells residing in the epithelial lining of the bronchi [7]. Comprehensive genomic analyses of SCLCs have reported loss-of-function alterations in tumor repressor genes *RB1* and *TP53* in ~ 90% of cases [8–12], in addition to a high prevalence of mutually exclusive amplification of *MYCL*, *MYC*, or *MYCN* (~ 20%), or mutations in *PTEN* (~ 8%) and *NOTCH* (~ 25%) family genes [8–12]. The cell types leading to SCLC have been more functionally characterized in terms of genetic derivation and origin than have the cells of many other tumors [13]. Conditional inactivation of *Rb1* and *Trp53* in lung epithelial cells (in an *RP* mouse model) led to the development of dysplastic precancerous lesions, which morphologically and genetically mimic human SCLC [14]. During the long latency (10–15 months) in the development of SCLC, the *RP* tumors were shown to acquire *Mycl* amplifications, *Nfib* amplifications, or *Pten* loss [15]. Inactivation of one allele of *Pten* in the *RP* background significantly accelerated tumorigenesis, after which *RP* mice became moribund at an average of ~ 13 months, compared with ~ 6 months in *Pten*^*lox/+*^*RP* and ~ 4 months in *Pten*^*lox/lox*^*RP* mice [16]. In addition to *Pten*, the conditional knockout of *Crebbp*, the constitutive expression of *Mycl*, or a stabilized *Myc* mutant (MycT58A) was shown to accelerate SCLC tumorigenesis in mice [15, 17].

The PI3K/AKT/mTOR pathway is closely associated with growth, survival, and the invasion of cancers. Phosphatidylinositol 3-kinases (PI3Ks) belong to the lipid kinase family, which can be divided into three main classes (Class I, II, and III), in accordance with their structural and substrate specificities [18, 19]. Class IA PI3Ks are composed of a heterodimer between a p110 catalytic subunit (PIK3CA, PIK3CB, or PIK3CD) and a p85 regulatory subunit [20]. Upon receiving extracellular activation signals via tyrosine kinase receptors, PI3Ks phosphorylate PIP2 to PIP3, the process of which is counteracted by PTEN. PIP3 recruits AKT to the plasma membrane for activation by PDK1, which initiates a subsequent signaling cascade for cell growth, enhanced metabolic activity, and cell survival [21]. Mammalian target of rapamycin (mTOR) is considered a class IV PI3K-related serine/threonine kinase composed of ataxia telangiectasia mutated (ATM), ataxia telangiectasia and Rad3 related (ATR), DNA-dependent protein kinase (DNA-PK), and mTOR [19]. mTOR exhibits two functionally distinct complexes: mTOR complex 1 (mTORC1) and mTOR complex 2 (mTORC2) [22]. mTORC1 is defined by the adaptor protein RPTOR (regulatory-associated protein of mTOR) and is rapamycin-sensitive, whereas mTORC2 has been observed associating with RICTOR (rapamycin-insensitive companion of mTOR) [23]. mTORC1 is well characterized as a major driver of cell growth by promoting cap-dependent translation through the phosphorylation of the ribosomal protein S6 kinases (P70S6K1 and P70S6K2) and the eukaryotic initiation factor 4E-binding proteins (4EBP1 and 4EBP2) [21, 24]. The function of mTORC2 is less characterized; however, it is known to activate AKT, which in turn phosphorylates and inhibits mTORC1 repressor TSC2 [21, 24].

Cancers that acquire a mutation/copy gain in PIK3CA or loss of PTEN have been implicated in enhanced sensitivity to inhibitors targeting the PI3K/AKT/mTOR pathway. However, the complexities of the pathway and corresponding feedback loops hamper clear predictions as to the response of tumors presenting these genomic features [25–28]. In the current study, we evaluated the sensitivity of 13 SCLC cell lines to inhibitors targeting PI3K, AKT, and mTOR. We determined that the degree of cell growth inhibition by dual PI3K and mTOR inhibitors in SCLCs was positively correlated with AKT phosphorylation levels at Thr308 and Ser473, but not correlated with PTEN levels or PI3KCA mutation/copy gain. These results suggest a potential strategy for the treatment of SCLC involving the combinational use of clinically approved PI3K and mTOR inhibitors.

## Methods

### Test compounds

MK-2206 (A10003), RAD001 (A10374), CAL-101 (A10172), LY294002 (A10547), and z-VAD-FMK (A12373) were purchased from AdooQ Biosciences. BAY 80-6946 (MedChemExpress, HY-15,346), BYL719 (Cayman Chemical, 16986), NVP-BGT226 (Cayman Chemical, 22142), Staurosporine (Cayman Chemical, 81590), and GSK2126458 (Cayman Chemical, 17377) were purchased from vendors.

### Culturing of cell lines

All SCLC cell lines, including NCI-H82, NCI-H446, NCI-H69, NCI-H211, NCI-H524, NCI-H526, NCI-H146, NCI-H187, NCI-H345, NCI-H841, NCI-H1930, NCI-H2171, and NCI-H2081, were obtained from American Type Culture Collection (ATCC). NCI-H345, NCI-H841, NCI-H2171, and NCI-H2081 cell lines were maintained in HITES medium [Dulbecco’s medium: Ham’s F12, 50:50 mix (Gibco), insulin 0.005 mg/ml, transferrin 0.01 mg/ml, sodium selenite 30 nM, hydrocortisone 10 nM, and beta-estradiol 10 nM] containing 5% fetal bovine serum (FBS, Hyclone) and penicillin–streptomycin antibiotics (Gibco). The remainder of the cell lines were cultured in RPMI1640 medium (Gibco) supplemented with 10% FBS and antibiotics. Cells were cultured for up to 8 weeks and used at passages of less than 16 from the initial source vial.

### Cell viability

Cells were seeded in 96-well plates (Corning) at densities optimized for each line (5000–10,000 cells per well). After 24 h, cells were treated with the designated compounds for 72 h. Cell viability was determined using PrestoBlue Cell Viability Reagent (ThermoFisher Scientific). Fluorescence was measured using a Perkin Elmer Wallac 1420 Victor2 plate reader. Cell viability was calculated by normalizing each blank-corrected fluorescence value to untreated cells. The computation of IC_50_ values was based on an eight-point titration performed in triplicate. Synergy between any two compounds was determined using SynergyFinder (https://synergyfinder.fimm.fi) based on the zero interaction potency (ZIP) model [29].

### RNAi

Small interfering RNAs (siRNAs) targeting *PTEN* (ThermoFisher Scientific, 4390771/Assay ID: n276787 and 4392420/Assay ID: s327), *CASP3* (Qiagen, FlexiTube siRNAs SI02661939 and SI02662450), or *CASP6* (Qiagen, FlexiTube siRNAs SI02654603 and SI03100041) were purchased from vendors. Two sets of siRNAs for one gene were mixed at a ratio of 1:1 for expression depletion. Cells were transfected with siRNAs using Lipofectamine™ RNAiMAX (ThermoFisher Scientific) in accordance with the manufacturer’s protocol.

### Immunoblotting

Cancer cells were collected from culture plates, washed using 1× phosphate-buffered saline (PBS), and lysed using RIPA buffer (50 mM HEPES, pH 7.3, 150 mM NaCl, 2 mM EDTA, 20 mM β-glycerophosphate, 0.1 mM Na_3_VO_4_, 1mM NaF, and 0.5 mM DTT) containing 0.5% NP-40 (Sigma-Aldrich), protease inhibitor cocktail (Roche Applied Science), and phosphatase inhibitor (Roche Applied Science). Cell lysates were then prepared in 1× Laemmli protein sample buffer, and boiled at 100 °C for 10 min. For xenograft tumors, tissues were dissected into small pieces, weighed, lysed in 1× Laemmli protein sample buffer under rigorous sonication, and boiled at 100 °C for 10 min. Lysates were separated using sodium dodecyl sulfate polyacrylamide gel electrophoresis (SDS-PAGE) and transferred to polyvinylidene fluoride (PVDF, Millipore) membranes. Membranes were blotted with primary antibodies which were diluted in 0.2% casein, 1% bovine serum albumin (BSA), or 5% nonfat milk. After overnight blotting at 4 °C, the membranes were washed using blotting buffer [0.2% casein in 1× PBS with 0.1% Tween^®^ 20 detergent (PBST) or 1× Tris-buffered saline with 0.1% Tween^®^ 20 detergent (TBST)]. Corresponding horse radish peroxidase (HRP)- or alkaline phosphatase (AP)-conjugated secondary antibodies (Sigma-Aldrich) were added. The blots were developed by chemiluminescence in accordance with the manufacturer’s protocols. Primary antibodies obtained from Cell Signaling were PTEN (#9188), AKT (#04-796), p-AKT(Thr308) (#13038), p-AKT(Ser473) (#9271), P70S6K (#2708), p-P70S6K(Thr389) (#9234), 4EBP1 (#9644), p-4EBP1(Thr37/Thr46) (#2855), PIK3CA (#4249), RICTOR (#9476), CASP6 (#9762), cleaved CASP3 (c-CASP3, #9661), cleaved CASP6 (c-CASP6, #9761), CASP7 (#12,827), and CASP8 (#4790). The other primary antibodies were RPTOR (abcam, ab40768), PARP-1 (abcam, ab32378), CASP3 (Genetex, GTX123678), GAPDH (Genetex, GTX100118), and β-ACTIN (Sigma-Aldrich, A1978).

### In vivo xenograft studies

NCI-H446 cells were prepared in cold 1× PBS, mixed with an equal volume of Matrigel^®^ Basement Membrane Matrix (Corning), and transplanted subcutaneously (at 1 × 10^6^ cells/mouse) into the right flank of 6- to 8-week-old male athymic nu/nu mice (BIOLASCO). The mice were housed in individually ventilated cages, which were maintained under 12 h light/dark cycles with controlled temperature and humidity. The size of the xenografted tumors was measured using a digital caliper (Goldsun Electronics Co.) and calculated in accordance with the following formula: tumor volume (mm^3^) = length × (width)^2^/2. Body weight and tumor size were measured at least twice a week. Mice were randomly assigned to control and treatment groups when the tumors reached 150–250 mm^3^ in size. The mice were orally administered with a vehicle (0.5% methylcellulose and 0.2% Tween-80 in ddH_2_O) or 1.5 mg/kg GSK2126458 [30] on a 5-on-2-off dosing regimen for 2 weeks. The animal protocol had been reviewed and approved by Institutional Animal Care and Use Committees (IACUC) of the National Health Research Institutes. The care and use of animals was conducted in accordance with regulations outlined by the Association for Assessment and Accreditation of Laboratory Animal Care (AAALAC).

### In vitro caspase reaction

NCI-H446 cells lysed in RIPA buffer were incubated with various quantities of recombinant human active Caspase-3 (CASP3, abcam, ab52101) or Caspase-6 (CASP6, abcam, ab52157) in 40 µL of 1× Caspase reaction buffer (50 mM HEPES, 50 mM NaCl, 0.1% CHAPS, 10 mM EDTA, 5% Glycerol, and 10 mM DTT) [31]. The reaction was performed at 37 °C for various durations ranging from 15 min to 3 h. Reactions were stopped by the addition of Laemmli protein sample buffer and boiling at 100 °C for 10 min, before undergoing SDS-PAGE and Western blot analyses.

### Statistical analysis

Statistical differences were analyzed using two-tailed Student’s t-test or two-way ANOVA. The criterion for significance (p-value) was set as described in the figures.

## Results

### AKT phosphorylation levels were positively correlated with the sensitivity of SCLC to PI3K and mTOR dual inhibition

**Fig. 1:**
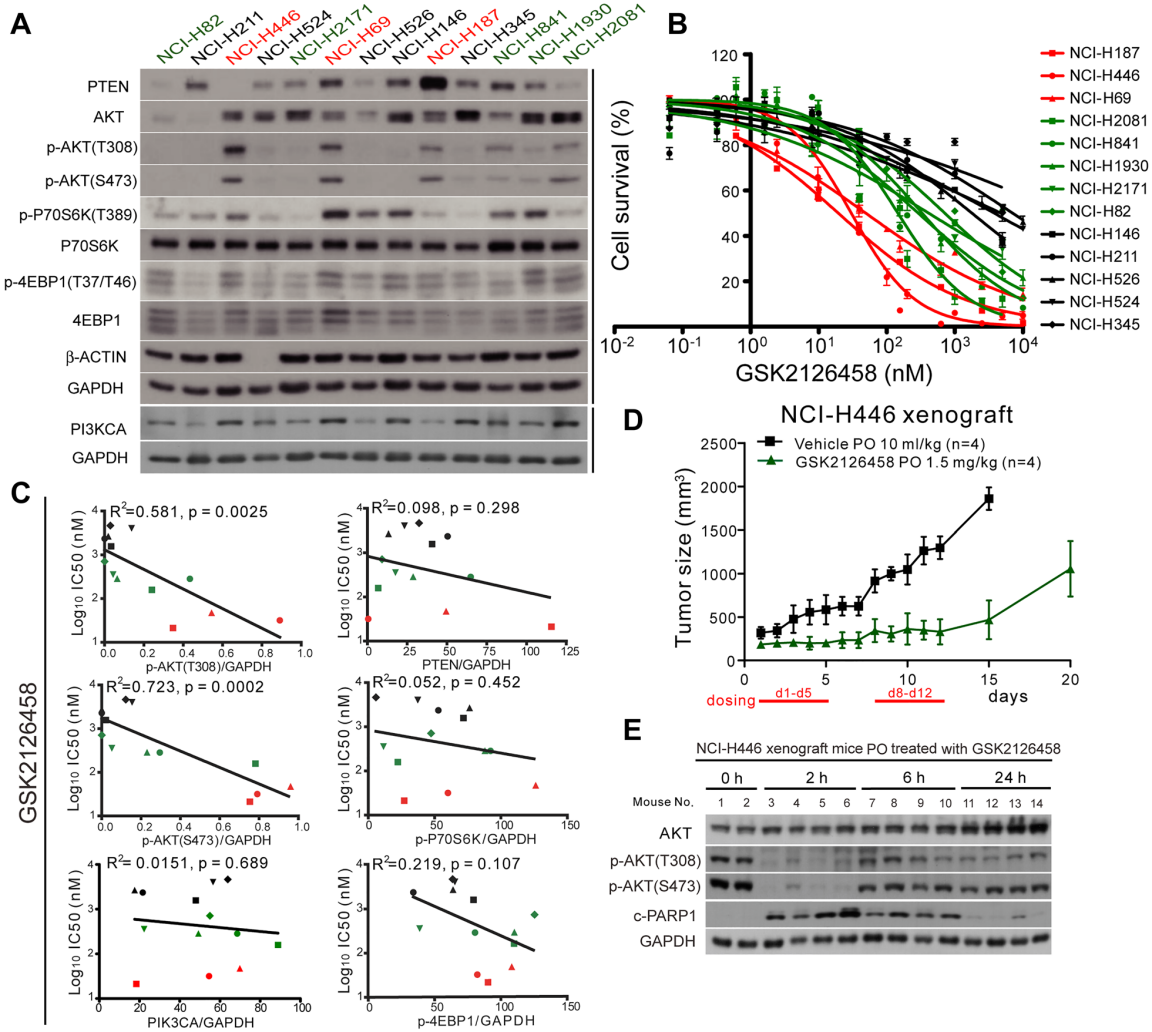
SCLC cell lines with high p-AKT were sensitive to PI3K/mTOR dual inhibition. **A** Western blot analysis showing the expression of proteins involved in the PI3K/AKT/mTOR pathway in 13 SCLC cell lines. **B** Concentration-response cell survival curves of SCLC cell lines treated using GSK2126458. **C** Correlation between survival response (IC_50_) and the levels of p-AKT(T308), p-AKT(S473), PIK3CA, PTEN, p-P70S6K, and p-4EBP1. Symbols of the cell lines are the same as those denoted in **B**. R^2^, coefficient of determination. **D** Tumor growth curve of xenografted NCI-H446 cells in nude mice. Mice were orally (PO) administered with vehicle (10 ml/kg) or GSK2126458 (1.5 mg/kg) when the tumor size reached approximately 200 mm^3^ using a 5-on-2-off cycle for 2 consecutive weeks. **E** Western blot analysis results showing the expression of AKT, p-AKT(T308), p-AKT(S473), cleaved PARP-1 (c-PARP1), and a GAPDH loading control in xenografted NCI-H446 tumors in mice oral administered with GSK2126458 (3.0 mg/kg). Tumors were harvested at 2, 6, and 24 h after drug treatment

The PI3K/AKT pathway is centrally involved in the transmission of mitogenic signals to mTOR. In examining the differential activation signaling of PI3K/AKT/mTOR in SCLCs, we first determined the phosphorylation levels of AKT(T308), AKT(S473), P70S6K(T389), and 4EBP1(T37/T46) as well as PTEN and PIK3CA in 13 SCLC cell lines (Fig. 1A). We then quantified protein phosphorylation inhibition and cell survival (i.e., IC_50_) in response to treatment with GSK2126458 (a highly potent inhibitor of PI3Kα/β/δ/γ and mTORC1/2) [32], MK-2206 (a highly selective inhibitor of AKT1/2/3), RAD001 (an mTOR inhibitor of FKBP12), or LY294002 (a broad-range PI3K inhibitor) (Fig. 1B and Additional file 1: Figure S1). Genomic features of the 13 SCLC cell lines, including mutation/copy number in PIK3CA and PTEN, are listed in Table 1.

**Table 1:** Genomic features of the 13 SCLC cell lines used in this study and their sensitivity to GSK2126458 treatment

Cell Line	PIK3CA mutation/copy number	PTEN mutation	Relative level of p-AKT (T308)/(S473)	GSK2126458 IC_50_ ± SD (nM)
NCI-H187	WT/1.87^3^	WT	0.348/0.753	21.2 ± 6.0
NCI-H446	WT/NA	Exon 4, 46 bp deletion from codon 82^1^	0.891/0.791	31.7 ± 13.8
NCI-H69	G106-R108 del./3.69^3^	WT	0.544/0.960	47.3 ± 19.2
NCI-H2081	WT/NA	Missense Mutation, C124F^2^	0.240/0.782	158.4 ± 98.8
NCI-H1930	WT/1.90^3^	WT	0.064/0.231	285.3 ± 74.4
NCI-H841	WT/copy number gain^4^	WT	0.434/0.295	329.6 ± 59.3
NCI-H2171	WT/1.28^3^	WT	0.043/0.051	358.8 ± 239.6
NCI-H82	WT/1.69^3^	WT	ND/ND	710.6 ± 204.4
NCI-H146	WT/1.74^3^	WT	0.033/0.021	1572.3 ± 388.4
NCI-H211	WT/1.54^3^	WT	ND/ND	2354.5 ± 973.3
NCI-H526	WT/2.19^3^	WT	0.019/ND	2665.9 ± 1979.0
NCI-H524	WT/1.66^3^	WT	0.137/0.154	4035.2 ± 455.9
NCI-H345	WT/3.45^3^	WT	0.029/0.122	4622.3 ± 1183.9

*WT* wild type, *NA* not available, *ND* not detectable

^1^See reference [51]

^2^Information retrieved from DepMap Portal (Broad Institute) https://depmap.org/portal/

^3^See reference [46]

^4^See reference [52]

Treatment with MK-2206 or LY294002 was shown to reduce the phosphorylation of AKT(T308) and AKT(S473), as previously reported (Additional file 1: Figure S1A); however, the survival of the 13 SCLC cells lines was unaffected at the concentration of 1.0 µM (Additional file 1: Figure S1B, C). Treatment with 1.0 µM RAD001 reduced survival by less than 20% in all SCLCs except for NCI-H446 (Additional file 1: Figure S1D). Most of the SCLC cell lines that expressed lower AKT phosphorylation [i.e., p-AKT(S473) level < 0.2, Table 1] remained > 50% viable after treatment with 1.0 µM GSK2126458 (IC_50_ > 1.0 µM, including NCI-H211, NCI-H524, NCI-H526, NCI-H146, and NCI-H345). Other SCLC cell lines expressing higher levels of p-AKT [i.e., p-AKT(S473) level > 0.7, Table 1], including NCI-H187, NCI-H446, and NCI-H69, were highly sensitive (IC_50_ < 100 nM) to treatment with GSK2126458 (Fig. 1B; Table 1). Note that the sensitivity of the SCLCs to GSK2126458 was strongly correlated with the level of AKT phosphorylation at T308 (R^2^ = 0.581, p = 0.0025) and S473 (R^2^ = 0.723, p = 0.0002) (Fig. 1C). In these SCLC cell lines, we did not observe correlations between the IC_50_ values of GSK2126458 and PIK3CA and PTEN levels or P70S6K and 4EBP1 phosphorylation levels (Fig. 1C).

GSK2126458 has previously demonstrated broad antitumor activity in preclinical studies across a broad panel of cancer cell lines [28]; however, the in vivo efficacy of GSK2126458 in treating SCLC has not been tested. Thus, we examined the growth inhibition activity of GSK2126458 in the NCI-H446 xenograft model, in which p-AKT was strong. Oral administration of 1.5 mg/kg GSK2126458 using a 5-on-2-off regimen for two weeks was shown to induce > 90% volume suppression of the xenograft tumors at day 12, compared to the vehicle control (Fig. 1D, p < 0.0001, t-test). We also examined the in vivo targeted inhibition of GSK2126458 in NCI-H446 xenograft tumors collected from untreated and drug-treated mice at 2, 6, and 24 h after dosing. p-AKT(T308) and p-AKT(S473) levels were significantly lower in xenograft tumors treated with GSK2126458 at 2 h after drug administration. This treatment also led to extensive cell apoptosis, as evidenced by PARP-1 cleavage (i.e., c-PARP1, Fig. 1E). These results demonstrate the efficacy of dual PI3K and mTOR inhibition in inducing cell apoptosis in xenograft SCLC tumors presenting strong p-AKT.

### Synergistic effects of PI3Kα and mTOR in limiting the survival of SCLCs presenting high p-AKT levels

It appears that the clinical trial of GSK2126458 may have been halted in phase I as a consequence of the high occurrence of grade ≥ 3 adverse events, including diarrhea, hyperglycemia, and skin rash [28]. Nonetheless, our findings suggest that PI3K and mTOR dual inhibition may be an effective therapeutic strategy for the treatment of SCLCs with high p-AKT. To date, five PI3K inhibitors have obtained FDA approval. These compounds inhibit specific isoforms of PI3K, including Idelalisib (i.e., CAL-101, a PI3Kδ inhibitor), Copanlisib (i.e., BAY 80-6946, a pan-class I PI3K inhibitor with subnanomolar IC_50_s against PI3Kα and PI3Kδ), Duvelisib (a dual inhibitor of PI3Kδ and PI3Kγ), Alpelisib (i.e., BYL719, an alpha-specific PI3K inhibitor), and Umbralisib (a dual inhibitor of PI3Kδ and CK1ε) [33–35]. Thus, we used these isoform-specific PI3K inhibitors to investigate the potential synergistic effects of PI3K-isoform and mTOR inhibition in SCLCs (Additional file 1: Figure S2A).

**Fig. 2:**
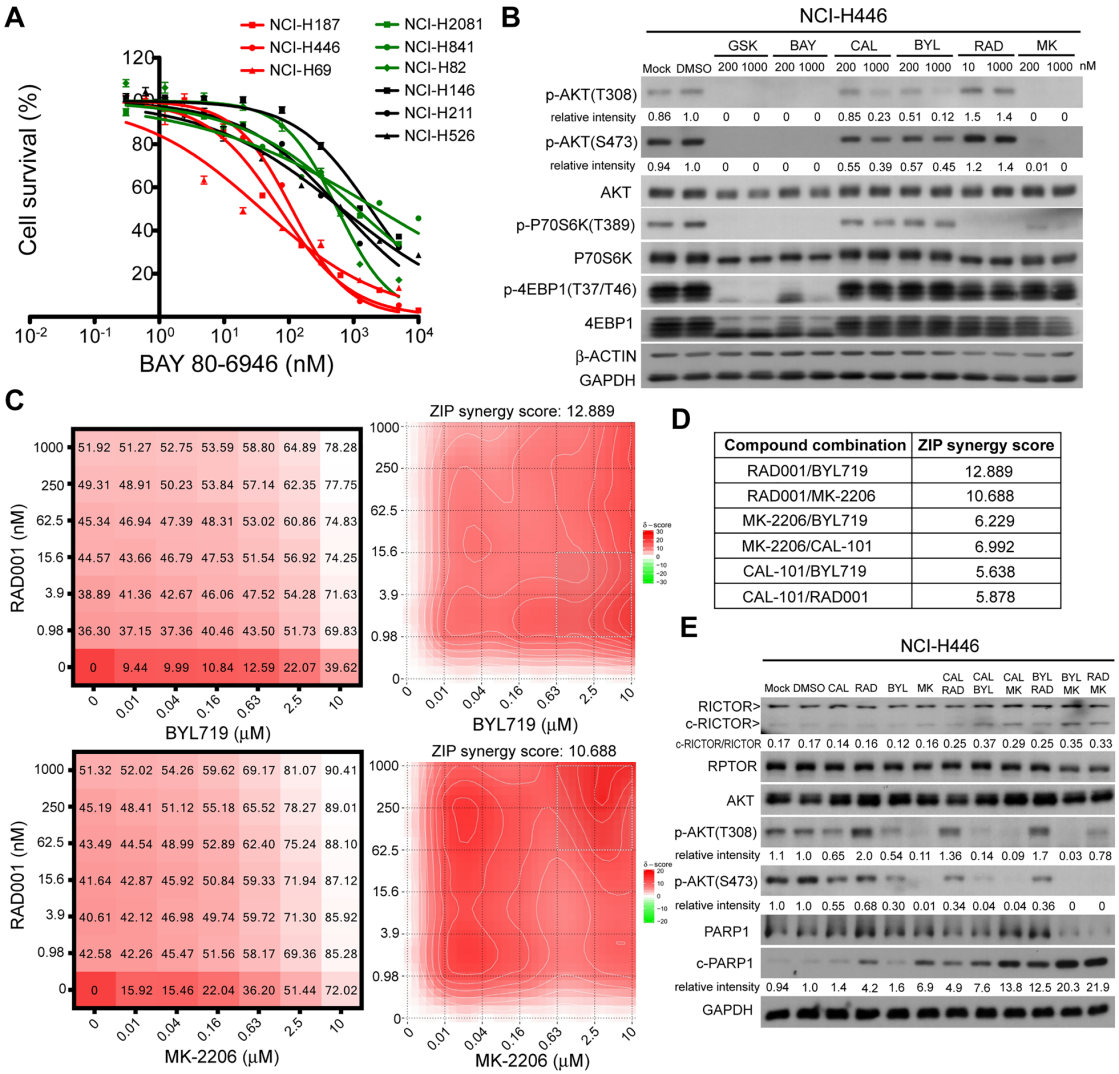
A combination of PI3K and mTOR inhibitors presented synergistic effects in limiting the survival of SCLC cells with high p-AKT. **A** Concentration-response cell survival curves of SCLC cell lines treated using BAY 80-6946. **B** Western blot analysis showing the expression of proteins involved in the PI3K/AKT/mTOR pathway in NCI-H446 cells treated with indicated concentrations of GSK2126458 (GSK), BAY 80-6946 (BAY), CAL-101 (CAL), BYL719 (BYL), RAD001 (RAD), or MK-2206 (MK) for 24 h. Levels of p-AKT(T308) and p-AKT(S473) were quantified and listed below the profiles. **C** Dose matrix of RAD001 in combination with BYL719 or MK-2206. Percent growth inhibition at each dose was assessed at 72 h after drug treatment (see left panel). Synergy distribution and synergy scores based on a zero interaction potency (ZIP) model (shown on the right). **D** List of ZIP scores presenting synergy of drug combinations (any two of CAL, BYL, RAD and MK) to the survival of NCI-H446 cells. **E** Western blot analysis showing the expression of the indicated proteins in NCI-H446 cells following single or combination treatment using GSK, BAY, CAL, BYL, RAD001, or MK-2206 at a concentration of 1.0 µM. The lower band under full-length RICTOR is cleaved RICTOR (c-RICTOR). The expression of c-PARP1 used to denote cell apoptosis. Levels of c-PARP1, c-RICTOR, c-RPTOR, p-AKT(T308) and p-AKT(S473) were quantified and listed below the profiles

Similar to the results of GSK2126458 treatment (Fig. 1B), the survival of high p-AKT SCLCs (i.e., NCI-H187, NCI-H446, and NCI-H69) was more sensitive to BAY 80-6946 than were low p-AKT SCLCs (Fig. 2A). As expected, BAY 80-6946 rendered p-AKT(T308) and p-AKT(S473) levels undetectable and led to a decrease in p-P70S6K and p-4EBP1 levels (Fig. 2B), which is an indication of mTOR inhibition. CAL-101 and BYL719 were both shown to reduce phosphorylation of p-AKT(T308) and p-AKT(S473) by > 50% at a concentration of 1.0 µM (Fig. 2B); however, SCLCs with high p-AKT were more sensitive to BYL719 than to CAL-101 (Additional file 1: Figure S2B).

We next measured the potential synergy between isoform-selective PI3K inhibitors (i.e. CAL-101 and BYL719), MK-2206, and RAD001 in SCLC survival via combination profiling. Dose matrices of increasing concentrations of any two of the four drugs were assessed in terms of their effects on cell viability. Drug synergy was evaluated using SynergyFinder (version 2.0), a web-based interactive analysis and visualization application [36]. A ZIP model was used to evaluate drug additivity and calculate a synergy score. A synergy score of greater than 10 suggests that any interaction between two drugs is likely to be synergistic [29]. Our results identified combinations of BYL719/RAD001 and MK-2206/RAD001 as synergistic with synergy scores of 12.889 and 10.688, respectively (Fig. 2C). Other combinations, including CAL-101/BYL719, CAL-101/RAD001, CAL-101/MK-2206, and MK-2206/BYL719, additively inhibited the survival of NCI-H446 (Fig. 2D and Additional file 1: Figure S2C).

Our Western blot results were in line with the synergy scores, indicating that RAD001 in combination with BYL719 or MK-2206 had a synergistic effect on the level of cleaved PARP-1 (c-PARP1), which is indicative of synergistically enhanced cell death. Combinations of CAL-101/BYL719, CAL-101/RAD001, CAL-101/MK-2206, RAD001/MK-2206, and MK-2206/BYL719 were also shown to increase c-PARP1 levels, compared to single drug treatments (Fig. 2E). These results indicate that the PI3K isoforms work differently with mTOR in terms of the survival of SCLCs with high p-AKT.

### Dual PI3K and mTOR inhibition induce proteolytic cleavage of RICTOR and RPTOR in SCLCs with elevated p-AKT expression levels

**Fig. 3:**
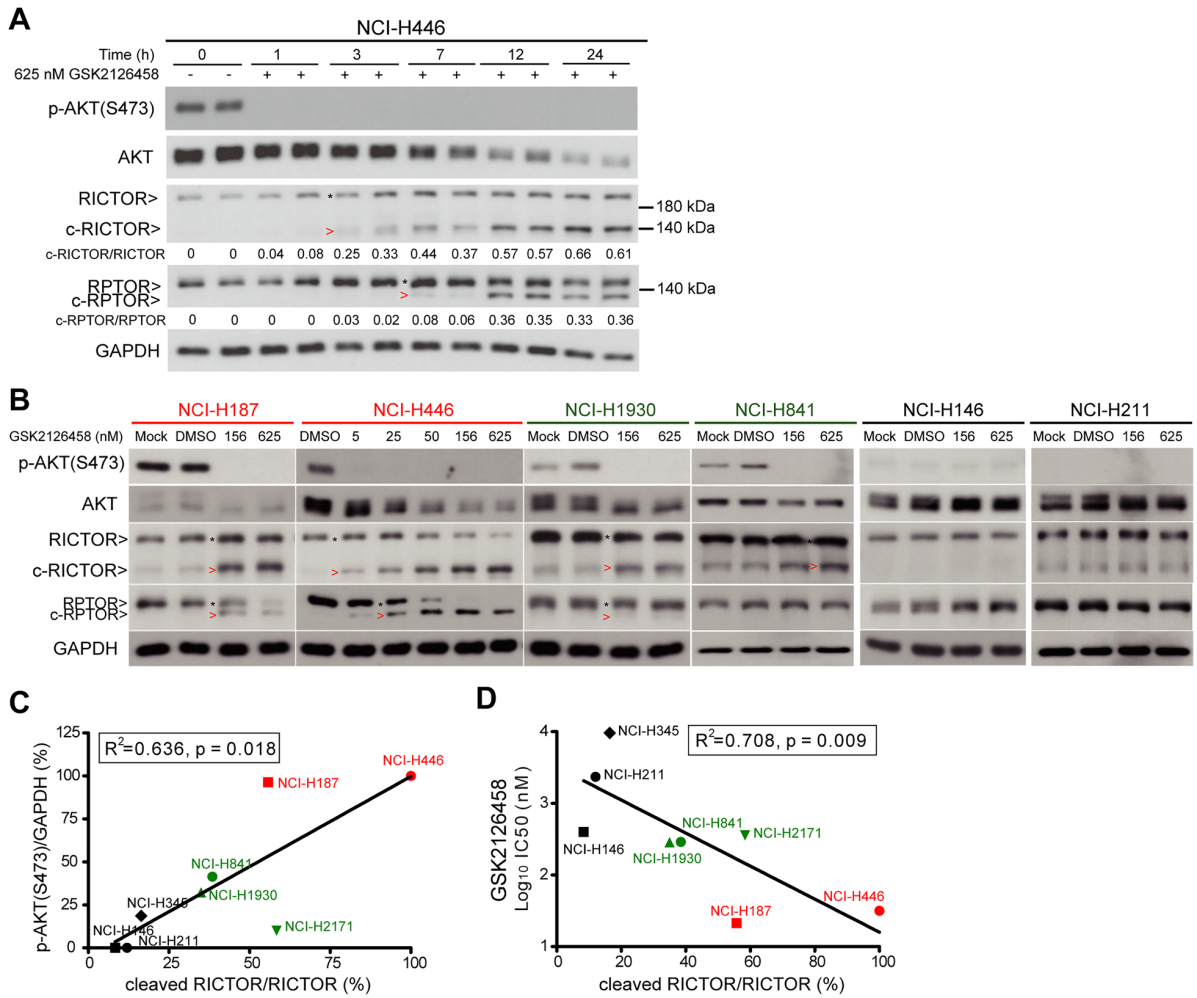
GSK2126458 induces proteolytic cleavages of RICTOR and RPTOR in SCLCs with high p-AKT. **A** Western blot analysis showing the time-dependent cleavages of RICTOR and RPTOR in NCI-H446 cells treated using GSK2126458 (625 nM). The expression level of p-AKT(S473) is shown to illustrate the efficacy of GSK2126458 in inhibiting AKT phosphorylation. The black stars denote full-length RICTOR/RPTOR, and the red arrowheads indicate cleaved RICTOR/RPTOR. Levels of c-RICTOR and c-RPTOR were quantified and listed below the profiles. **B** Western blot analysis illustrating the expression of AKT, p-AKT(S473), RICTOR, and RPTOR in SCLC cell lines treated using GSK2126458 at indicated concentrations for 24 h. The black stars denote full-length RICTOR/RPTOR, and the red arrowheads indicate cleaved RICTOR/RPTOR. **C** Correlation between the level of p-AKT(S473) normalized to GAPDH and the ratio of c-RICTOR in eight SCLC cell lines treated as described in (**A**). **D** Correlation between the survival response (i.e., IC_50_) of GSK2126458 and the ratio of c-RICTOR in SCLC cell lines. The coefficient of determination (R^2^) in each curve was calculated and presented in the figure

We described above that the simultaneous inhibition of PI3K and mTOR (or AKT and mTOR) reduced the viability of SCLC cells with high p-AKT levels (Figs. 1 and 2). Note that a lower band (approximately 140 kDa) appeared below the full-length RICTOR, the intensity of which coincided with c-PARP1 levels (Fig. 2E). The proteolytic cleavage of RICTOR was consistently observed in NCI-H446 treated with the PI3K/mTOR dual inhibitor GSK2126458 and the pan-class I PI3K inhibitor BAY 80-6946. Cleaved RICTOR (c-RICTOR) was observed as early as 3 h after treatment with GSK2126458 or BAY 80-6946, and the level was found to increase with time (Fig. 3A and Additional file 1: Figure S3A). Treatment using GSK2126458 or BAY 80-6946 was also shown to induce the proteolytic cleavage of RPTOR (c-RPTOR), the molecular size of which was approximately 130 kDa. Whereas the BYL719 and RAD001 combination abolished p-AKT(S473) and induced RICTOR cleavage at 1 h post drug treatment, the p-AKT(S473) signals resumed after 3 h. Moreover, c-RPTOR levels did not vary considerably between 3 and 24 h after drug treatment (Additional file 1: Figure S3A). These results suggest that incomplete blockading of PI3K and/or mTOR may reactivate the signaling pathway, which could be attributed to mTORC2 activation via p70S6K-IRS1 negative feedback, thereby limiting their efficacy [37, 38].

We next investigated whether p-AKT levels play a role in the proteolytic cleavage of RICTOR and RPTOR, which was achieved by shutting down PI3K/mTOR signaling using GSK2126458. Our results revealed that the degree of GSK2126458-induced RICTOR proteolytic cleavage was positively correlated with the level of p-AKT(S473) (R^2^ = 0.636, p = 0.018, Fig. 3B, C) and inversely correlated with IC_50_ (R^2^ = 0.708, p = 0.009, Fig. 3D). The c-RPTOR signal was less pronounced in SCLCs expressing moderate p-AKT levels (i.e., NCI-H1930 and NCI-H841) than in those expressing high p-AKT levels (i.e., NCI-H187 and NCI-H446). To verify that GSK2126458-induced RICTOR and RPTOR proteolytic cleavages were on-target effects, we measured the level of RICTOR and RPTOR in NCI-H446 respectively treated with BAY 80-6946 and another dual PI3K/mTOR inhibitor (NVP-BGT226). Consistent with our observations pertaining to GSK2126458-treated cells, we observed an increase in the level of RICTOR/RPTOR cleavage with an increase in the concentrations of the inhibitors (Additional file 1: Figure S3B). Note that the extent of BAY 80-6946-induced cleavage of RICTOR and RPTOR in high p-AKT SCLCs (i.e. NCI-H446 and NCI-H187) exceeded that in SCLCs with medium- (i.e., NCI-H841) or low- (i.e., NCI-H211) p-AKT (Additional file 1: Figure S3C). These results suggest that the proteolytic cleavage of RICTOR and RPTOR induced by inhibition of pan-class I PI3Ks and/or mTORs was associated with p-AKT(S473) levels.

### PTEN depletion sensitizes SCLC to PI3K/mTOR inhibition-induced cell death and proteolytic cleavage of RICTOR

**Fig. 4:**
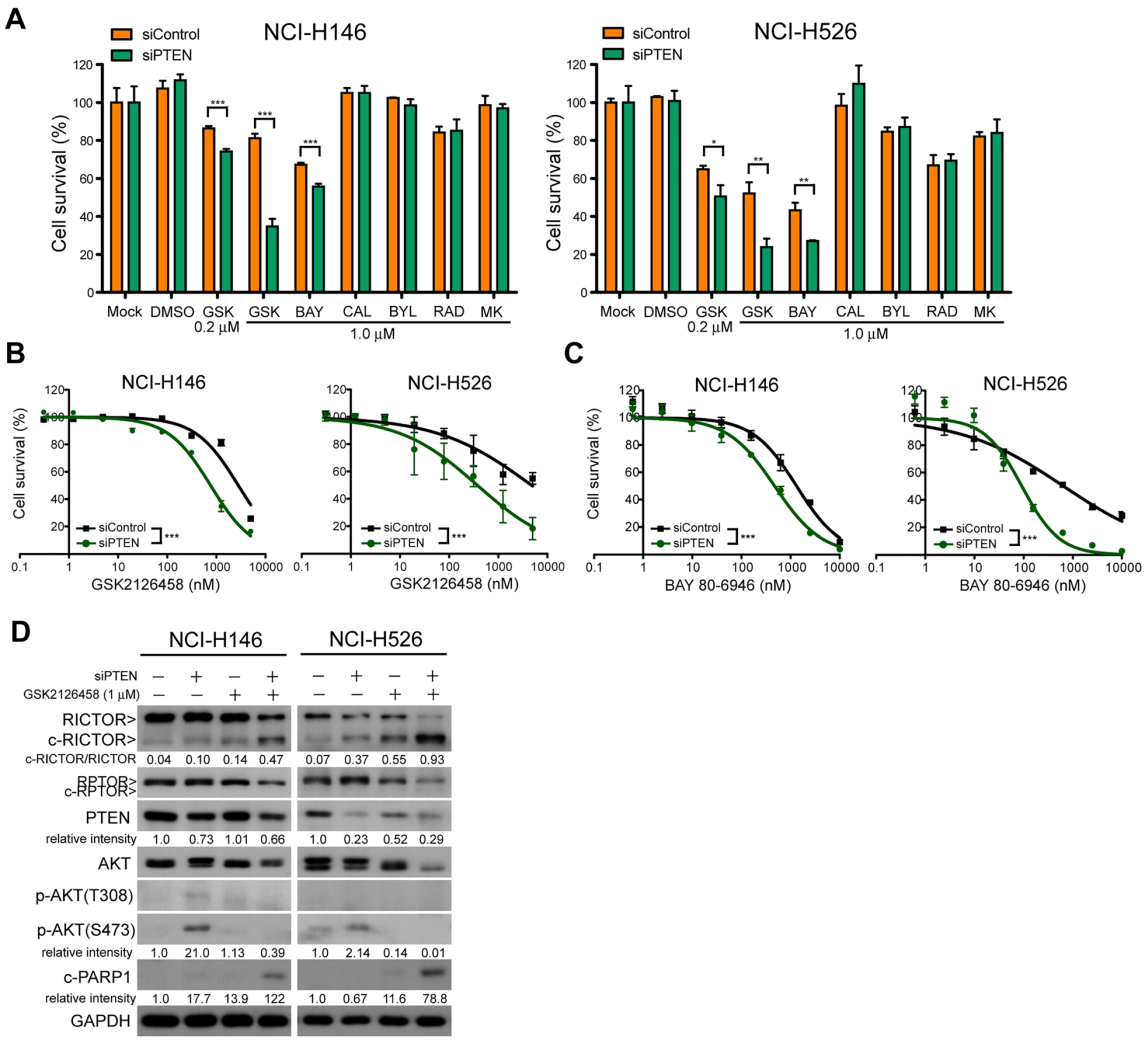
PTEN contributes to the survival of low p-AKT cells treated using PI3K/mTOR inhibitors. **A** Percent survival of NCI-H146 and NCI-H526 in response to treatment using the indicated compounds for 72 h. *p < 0.05; **p < 0.01; ***p < 0.001 (t-test). **B**, **C** Concentration-response cell survival curves of NCI-H146 and NCI-H526 cells pretreated using siRNAs targeting *PTEN* for 48 h, followed by treatment using GSK2126458 (**B**) or BAY 80-6946 (**C**) for an additional 72 h. ***p < 0.001 (two-way ANOVA). **D** NCI-H146 and NCI-H526 cells were first transfected with siRNAs targeting *PTEN* for 48 h. After another 12 h treatment using GSK2126458 (1.0 µM), cells were harvested and subjected to Western blot analysis using the indicated antibodies. Levels of c-RICTOR, c-PARP1, PTEN and p-AKT(S473) were quantified and listed below the profiles. The results indicated that c-RICTOR and c-PARP1 were increased in GSK2126458-treated cells depleted for *PTEN*

PTEN is a protein phosphatase that antagonizes the kinase activity of PI3K [37]. Having determined that the protein level of PTEN was not associated with the viability of SCLCs under dual PI3K/mTOR inhibition (Fig. 1C), we next sought to determine whether PTEN-mediated AKT dephosphorylation was correlated with the viability of SCLCs under PI3K and/or mTOR inhibition. In NCI-H146 and NCI-H526 cells (in which p-AKT levels were nearly undetectable), the depletion of PTEN significantly reduced the viability of cells under treatment with GSK2126458 or BAY 80-6946 (Fig. 4A–C). On the other hand, PTEN deletion did not alter survival rates in the two cell lines following treatment with CAL-101, BYL719, MK-2206, or RAD001 (Fig. 4A).

Western blot results verified that the depletion of PTEN elevated the levels of p-AKT(S473) and p-AKT(T308) (Fig. 4D). Under PTEN depletion, treatment with GSK2126458 eliminated AKT phosphorylation and promoted cell apoptosis, as evidenced by elevated c-PARP1 levels. Furthermore, the depletion of PTEN increased proteolytic cleavage of RICTOR, compared to PTEN-intact NCI-H146 and NCI-H526 cells (Fig. 4D). These results indicate that PTEN antagonized dual PI3K/mTOR inhibition-induced cell apoptosis in SCLCs with low intrinsic p-AKT levels.

### RICTOR and RPTOR are respective direct substrates of CASP6 and CASP3

**Fig. 5:**
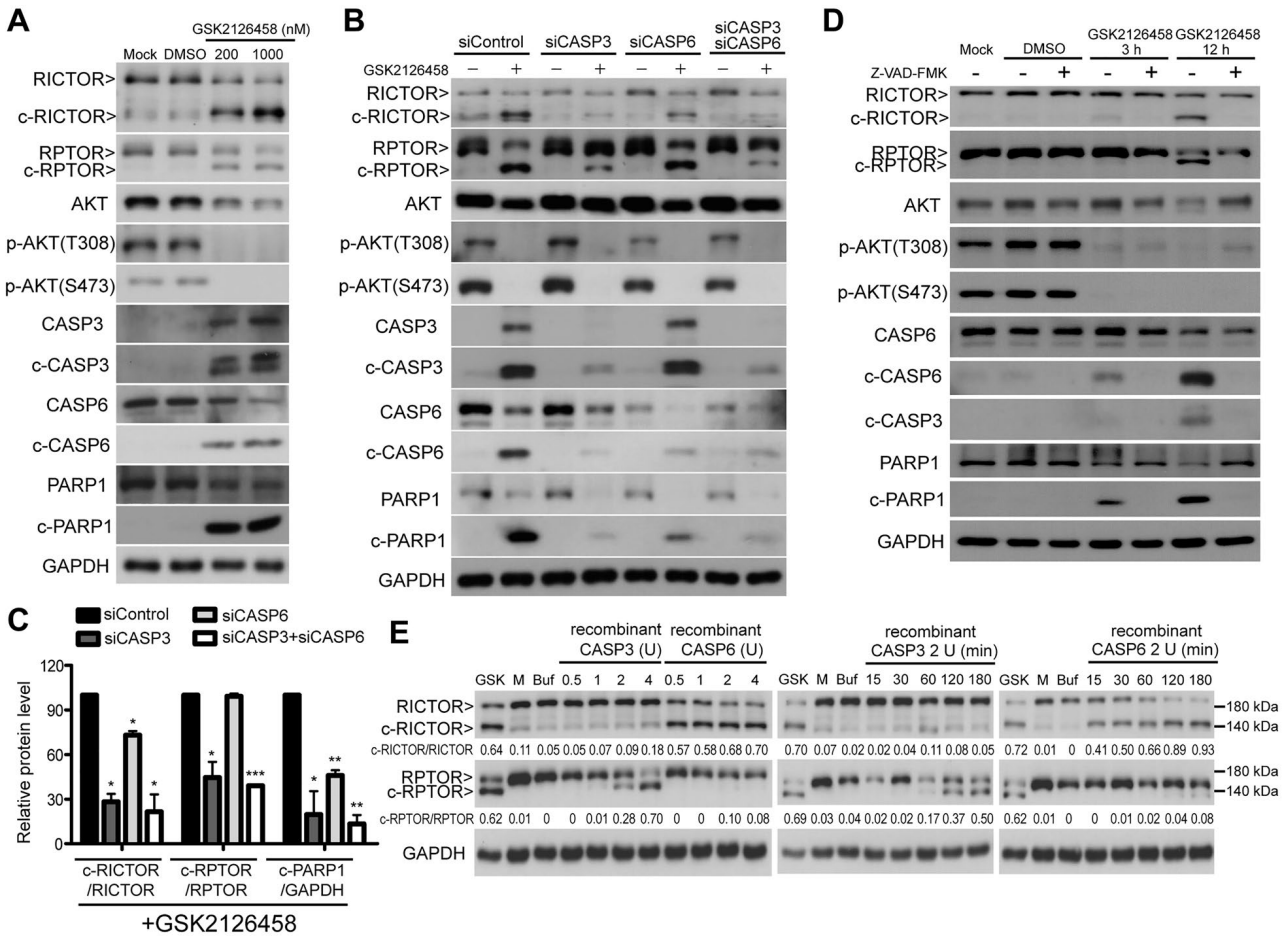
Cleavages of RICTOR and RPTOR by caspases upon PI3K/mTOR dual inhibition. **A** Western blot analysis of RICTOR and RPTOR cleavages and the activation of CASP3 and CASP6 in NCI-H446 cells treated using GSK2126458 (200 and 1000 nM) for 12 h. Cleavage of PARP1 (c-PARP1) and a loading control GAPDH were monitored and compared. **B** NCI-H446 cells were first transfected with siRNAs targeting either or both CASP3 and CASP6 for 48 h. After another 12 h of GSK2126458 treatment (1.0 µM), cells were harvested and subjected to Western blot analysis using the indicated antibodies. **C** Quantification for the ratios of c-RICTOR, c-RPTOR, and c-PARP1 in Western blot experiments of (**B**) (error bars are mean ± SD from duplicated experiments; *p < 0.05; **p < 0.01; ***p < 0.001, t-test). **D** Effects of z-VAD-fmk on the GSK2126458-induced cleavage of RICTOR and RPTOR in NCI-H446 cells. Cells were treated using GSK2126458 with or without z-VAD-FMK (10 µM) for 3 or 12 h. **E** NCI-H446 cell lysates were incubated with various quantities of recombinant CASP3 or CASP6 (0.5, 1, 2, and 4 U) for 15, 30, 60, 120, and 180 min. Following the enzymatic reaction, cleavages of RICTOR and RPTOR were obtained using Western blot analysis. Levels of c-RICTOR and c-RPTOR were quantified and listed below the profiles. The molecular sizes of cleaved RICTOR and RPTOR in NCI-H446 cells treated with GSK2126458 (GSK) are presented for comparison

To identify the caspase(s) that could be implicated in the cleavage of RICTOR and RPTOR, we first examined GSK2126458-induced caspase activation in NCI-H446. Among the caspases examined in this study (CASP3, CASP6, CASP7, and CASP8), we observed the expression of cleaved CASP3 (i.e., c-CASP3) and cleaved CASP6 (i.e., c-CASP6) (Fig. 5A and Additional file 1: Figure S4) was significantly elevated compare to the other caspases. In this experiment, Staurosporine (STS) was used as a positive control for cell apoptosis. Note that the molecular sizes of c-RICTOR and c-RPTOR induced by GSK2126458 were similar to those in STS-induced apoptotic cells (Additional file 1: Figure S4).

Using RNAi, we noted the depletion of CASP3 ameliorated GSK2126458-induced cleavage of RICTOR and RPTOR. On the other hand, the depletion of CASP6 ameliorated the cleavage of RICTOR but not of RPTOR. The cleavage level of RICTOR was further ameliorated by the dual depletion of CASP3 and CASP6 (Fig. 5B, C). The GSK2126458-elicited proteolytic cleavage of RICTOR and RPTOR can be eliminated through the addition of z-VAD-FMK, a general caspase inhibitor (Fig. 5D). These results suggest that the activation of caspases, including CASP3 and CASP6, is responsible for the proteolytic processing of RICTOR and RPTOR.

We next sought to determine whether RICTOR and RPTOR are direct substrates of protein caspases. This was achieved by incubating NCI-H446 lysates with recombinant CASP3 and CASP6 (Fig. 5E). We then compared the degree of RICTOR and RPTOR cleavages in cells treated with multiple dosages and corresponding time durations to elucidate the specificity of the recombinant caspases to substrates. RICTOR could be efficiently processed by CASP6 into a ~ 140 kDa protein, whereas RPTOR was more responsive to the enzymatic activity of CASP3, resulting in a cleaved form of ~ 130 kDa (Fig. 5E). The molecular sizes of c-RICTOR and c-RPTOR processed by recombinant CASP6 and CASP3 were similar to those of c-RICTOR and c-RPTOR in GSK2126458-treated cells. Researchers have previously reported on caspase-mediated cleavages of RICTOR and RPTOR following treatment with stimuli, such as the death ligand or small molecules [31, 38]. Martin et al. reported that the death signal-induced proteolytic cleavage of RPTOR was primarily mediated by CASP6, whereas Zhao et al. reported that RICTOR was a direct substrate of CASP3 [31, 38]. In the current study, RICTOR and RPTOR could both be processed by recombinant CASP3 and CASP6; however, RICTOR was more sensitive to CASP6 and RPTOR appeared more sensitive to CASP3, under the same experimental conditions (Fig. 5E). Taken together with the RNAi data (Fig. 5B, C), these results indicate that in high p-AKT SCLC cells, the dual inhibition of PI3K and mTOR can induce cell death, accompanied by CASP6- and CASP3-mediated proteolytic cleavage of mTOR companion proteins.

## Discussion

This study led us to devise a strategy by which to select SCLC patients who are more likely to benefit from isoform-specific PI3K and mTOR inhibitors already in clinical use. Inhibition of both PI3K and mTOR induced apoptosis in SCLCs with acquired high p-AKT levels, in conjunction with CASP6- and CASP3-mediated proteolytic cleavages of mTOR companion proteins RICTOR and RPTOR. The fact that the combined targeting of mTOR and PI3Kα was more effective than inhibiting mTOR and PI3Kδ suggests that PI3Kα-mediated signaling is more important to cell survival in SCLC cell lines with high p-AKT under mTOR inhibition.

Patients with lung adenocarcinomas presenting well-defined genomic alterations benefit greatly from genotype-directed therapies; however, researchers have yet to identify actionable targeted therapy strategies for SCLC. Note that the repurposing of existing drugs, such as tyrosine kinase inhibitors, Hedgehog signaling inhibitors, anti-apoptotic inhibitors, and anti-angiogenesis agents, has not had a meaningful clinical impact in treating SCLCs [13, 39, 40]. Gene mutations (e.g., *PIK3CA*, *PTEN*, and *RICTOR*) and activation of the PI3K/AKT/mTOR have been reported in genomic and proteomic profiling studies of SCLC tumor samples; however, there have been notable discrepancies in the results, due perhaps to relatively small sample sizes, differences in population (e.g., race, naïve or chemo-resistant) and sequencing techniques [8, 10, 12, 41]. Nevertheless, the effectiveness of *PTEN* inactivation in accelerating SCLC in genetic mutant mouse models suggests that the PTEN-associated signaling pathway is a strong therapeutic pathway for SCLC, as long as predictive biomarkers can be identified [15, 16, 40]. In the current study, we observed notable differences in PTEN levels among SCLC cell lines; however, there was an absence of correlation between PTEN levels (Fig. 1A) and the degree of cellular sensitivity to dual PI3K/mTOR inhibitors (Fig. 1C). Nonetheless, *PTEN* knock-down increased the expression of p-AKT as well as the likelihood of cell death induced by dual PI3K/mTOR inhibitors in SCLC cell lines with low p-AKT levels (i.e., NCI-H146 and NCI-H526) (Fig. 4D). These results supported the notion that PTEN-mediated AKT dephosphorylation is important to the survival of SCLCs when the activities of PI3Ks and mTORs are blocked.

Constitutive activation of PI3K activity has been well recognized in a subset of SCLCs, and the inhibition of PI3K/AKT signaling has been shown to block growth and promote apoptosis in SCLCs activated in AKT [42, 43]. Two pioneering works published when only an early-generation PI3K inhibitor (LY294002) was available suggested that LY294002 at a high molar concentration is required to erase AKT phosphorylation [42], and the cancer cell growth inhibition efficiency was suboptimal [43]. Moore et al. reported that the administration of LY294002 at a concentration of 10 µM reduced the growth of NCI-H69 cells by ~ 25%; however, the administration of Rapamycin (20 nM) in conjunction with LY294002 (10 µM) inhibited NCI-H69 cell growth by ~ 50%, which suggests that PI3Ks and mTORC1 may act synergistically in their effects on SCLC survival. Umemura et al. demonstrated that the survival of cisplatin-resistant SCLC cell lines could be suppressed using the new PI3K/mTOR dual inhibitor (BEZ235); however, not all SCLC cell lines harboring alterations in the PI3K/AKT/mTOR pathway exhibited the same degree of sensitivity to BEZ235 [10]. BEZ235 failed in subsequent clinical trials on advanced renal cell carcinoma, due to a high frequency of grade 3–4 adverse (~ 50%). In another study, BEZ235 treatment targeting advanced pancreatic neuroendocrine tumors and other solid tumors was discontinued in 39% of cases [44]. Similar adverse effects have been reported in other studies on dual PI3K/mTOR inhibitors, including GSK2126458, VS-5584, and GDC-0980. These on-target adverse effects raise doubts as to whether a full blockade of PI3Ks and mTORs is clinically applicable [28, 44]. In the current study, we examined the effects of clinically approved pan-class I and isoform-selective PI3K inhibitors on the survival of SCLCs. This led to the discovery of potential treatment strategies involving BAY 80-6946 alone or BYL719 and RAD001 in combination for SCLC (Fig. 2), which is a disease indication that has not previously been clinically investigated for these drugs. Similar to GSK2126458, the clinically approved pan-class I PI3K inhibitor BAY 80-6946 reduced viability and induced RICTOR/RPTOR cleavage in high p-AKT cells (Fig. 2A and Additional file 1: Figure S3). BYL719 and RAD001 in combination induced a feedback reactivation of PI3K/mTOR (Additional file 1: Figure S3A) and survival signaling soon after drug treatment; however, this could be overcome by adjusting dosing frequency of the drugs in clinical design. The availability of clinical toxicology results from these FDA-approved PI3K inhibitors would facilitate the design of clinical plans pertaining to the use of PI3K inhibitors for the treatment of SCLC, particularly in terms of working dosages and adverse effects.

The observed activation of PI3K/AKT/mTOR signaling and corresponding increase in AKT phosphorylation in non-small cell lung cancer (NSCLC) tumor specimens (50–73%) was associated with poor disease prognosis [45]. The constitutive stimulation of this pathway could be attributed to genetic mutations/amplifications of *PIK3CA* (14–25%), mutations/loss of *PTEN* (24–44%), and/or genetic mutations in their upstream regulators *EGFR* (10–20%) and *KRAS* (8–21%) [45–48]. Agents that target this pathway could potentially shut down survival signaling in NSCLC; however, early phase clinical trials using specific PI3K and/or mTOR inhibitors have yielded disappointing results [47]. The non-exclusive genetic alterations in *PIK3CA* and *EGFR* or *KRAS* have amplified complications associated with the clinical use of PI3K/mTOR inhibitors to NSCLC. Unlike NSCLC, it appears that *EGFR* and *KRAS* mutations are extremely rare in SCLC [9, 49]. Differences between NSCLC and SCLC in terms of genetic background could affect the clinical benefits of using PI3K/mTOR pathway inhibitors.

Our results revealed that inhibiting PI3K, AKT, or mTOR signaling induced caspase-mediated proteolytic cleavage of RICTOR and/or RPTOR, albeit to varying degrees (Figs. 2, 3, 4 and 5). The caspase-mediated cleavage of proteins basically ensures the irreversible commitment of cells to apoptosis; therefore, it appears that the proteolytic cleavage of RICTOR and RPTOR could ensure the blockade of the survival signals mediated via mTORC1 and mTORC2. This would prevent intrinsic and acquired resistance mechanisms, which would otherwise limit the therapeutic efficacy of small-molecule inhibitors targeting this pathway. Many of the drugs targeting the PI3K pathway are currently under clinical evaluation; however, only one phase I clinical trial (NCT02069158) is currently investigating the potential use of the dual PI3K/mTOR inhibitor PF-05212384 in combination with paclitaxel and carboplatin for the treatment of SCLC [50]. Our findings in this study using preclinical cell and animal models provide possible avenues by which to accelerate the development of SCLC therapeutic strategies using PI3K inhibitors, which are already in clinical use.

## Supplementary Information

**Additional file 1: Figure S1.** Cell survival of SCLC cell lines treated using PI3K, AKT, or mTOR inhibitors. A, Western blot analysis showing the expression of proteins involved in the PI3K/AKT/mTOR pathway in NCI-H446 cells treated using RAD001, Wortmannin, MK-2206, LY294002, and GSK2126458 at indicated concentrations for 24 h. B–D, Percent survival of the thirteen SCLC cell lines in response to treatment using MK-2206 (B), LY294002 (C), and RAD001 (D). **Figure S2.** Cell survival of SCLC cell lines treated using isoform-selective PI3K inhibitors. A, Compound name and major molecular target of the PI3K inhibitors. B, Percent survival of SCLC cell lines in response to treatment using CAL-101 or BYL719. C, Dose matrix of drug combinations between any two of the following compounds: MK-2206, BYL719, RAD001, and CAL-101. Percent growth inhibition at each dose assessed after drug treatment for 72 h was shown on left. Synergy distribution and synergy scores based on a ZIP model were shown on right. **Figure S3.** Proteolytic cleavages of RICTOR and RPTOR in NCI-H446 cells treated using PI3K/mTOR inhibitors. A, Western blot analysis showing the time-dependent cleavages of RICTOR and RPTOR in NCI-H446 cells treated using BAY 80-6946 (1 μM) or BYL719 (10 μM) and RAD001 (1 μM) in combination. B, Western blot analysis showing the expression of AKT, p-AKT(S473), RICTOR, and RPTOR in NCI-H446 cells treated using BAY 80-6946 or NVP-BGT226 at indicated concentrations for 24 h. C, Western blot analysis showing the expression of AKT, p-AKT(S473), RICTOR, and RPTOR in high p-AKT (NCI-H446 and NCI-H187), medium p-AKT (NCI-H841), and low p-AKT (NCI-H211) cells treated using BAY 80-6946 at indicated concentrations for 24 h. The expression levels of c-RICTOR and c-RPTOR were quantified and listed below the profiles in (A), (B) and (C). **Figure S4.** Activation of caspases in high p-AKT cells via dual PI3K/mTOR inhibition. Western blot analysis showing the cleavage of RICTOR and RPTOR and the expression of caspases in NCI-H446 cells treated using Staurosporine (STS, 2 μM) or GSK2126458 (200 and 1000 nM) for 7 h.

## Data Availability

All data generated in this study have been included in the article and additional files.
