# Supplementary material for: AKT phosphorylation as a predictive biomarker for PI3K/mTOR dual inhibition-induced proteolytic cleavage of mTOR companion proteins in small cell lung cancer
Source: Cell Biosci. 2022 Aug 2;12:122. doi: 10.1186/s13578-022-00862-y (PMC9344631; doi:10.1186/s13578-022-00862-y)
Supplement: Supplementary file 1 — Additional file 1: Figure S1. Cell survival of SCLC cell lines treated using PI3K, AKT, or mTOR inhibitors. A, Western blot analysis showing the expression of proteins involved in the PI3K/AKT/mTOR pathway in NCI-H446 cells treated using RAD001, Wortmannin, MK-2206, LY294002, and GSK2126458 at indicated concentrations for 24 h. B–D, Percent survival of the thirteen SCLC cell lines in response to treatment using MK-2206 (B), LY294002 (C), and RAD001 (D). Figure S2. Cell survival of SCLC cell lines treated using isoform-selective PI3K inhibitors. A, Compound name and major molecular target of the PI3K inhibitors. B, Percent survival of SCLC cell lines in response to treatment using CAL-101 or BYL719. C, Dose matrix of drug combinations between any two of the following compounds: MK-2206, BYL719, RAD001, and CAL-101. Percent growth inhibition at each dose assessed after drug treatment for 72 h was shown on left. Synergy distribution and synergy scores based on a ZIP model were shown on right. Figure S3. Proteolytic cleavages of RICTOR and RPTOR in NCI-H446 cells treated using PI3K/mTOR inhibitors. A, Western blot analysis showing the time-dependent cleavages of RICTOR and RPTOR in NCI-H446 cells treated using BAY 80-6946 (1 μM) or BYL719 (10 μM) and RAD001 (1 μM) in combination. B, Western blot analysis showing the expression of AKT, p-AKT(S473), RICTOR, and RPTOR in NCI-H446 cells treated using BAY 80-6946 or NVP-BGT226 at indicated concentrations for 24 h. C, Western blot analysis showing the expression of AKT, p-AKT(S473), RICTOR, and RPTOR in high p-AKT (NCI-H446 and NCI-H187), medium p-AKT (NCI-H841), and low p-AKT (NCI-H211) cells treated using BAY 80-6946 at indicated concentrations for 24 h. The expression levels of c-RICTOR and c-RPTOR were quantified and listed below the profiles in (A), (B) and (C). Figure S4. Activation of caspases in high p-AKT cells via dual PI3K/mTOR inhibition. Western blot analysis showing the cleavage of RICTOR and RPTOR and the expre [file 13578_2022_862_MOESM1_ESM.docx]

**AKT phosphorylation as a predictive biomarker for PI3K/mTOR dual inhibition-induced proteolytic cleavage of mTOR companion proteins in small cell lung cancer**

Ming-Chun Hung^1#^, Wan-Ping Wang^1#^, and Ya-Hui Chi^1,2*^

^1^Institute of Biotechnology and Pharmaceutical Research, National Health Research Institutes, Zhunan, Miaoli County 35053, Taiwan.

^2^Graduate Institute of Biomedical Sciences, China Medical University, Taichung 40402, Taiwan.

**^#^Hung and Wang contributed equally to this work**

**^*^Corresponding author.** Ya-Hui Chi, 35 Keyan Road, Zhunan, Miaoli County 35053, Taiwan, Ph: +886-37-206166ext35718/Fax: +886-37-586456, Email: [ychi@nhri.org.tw](mailto:ychi@nhri.org.tw)

**Additional file 1**

**
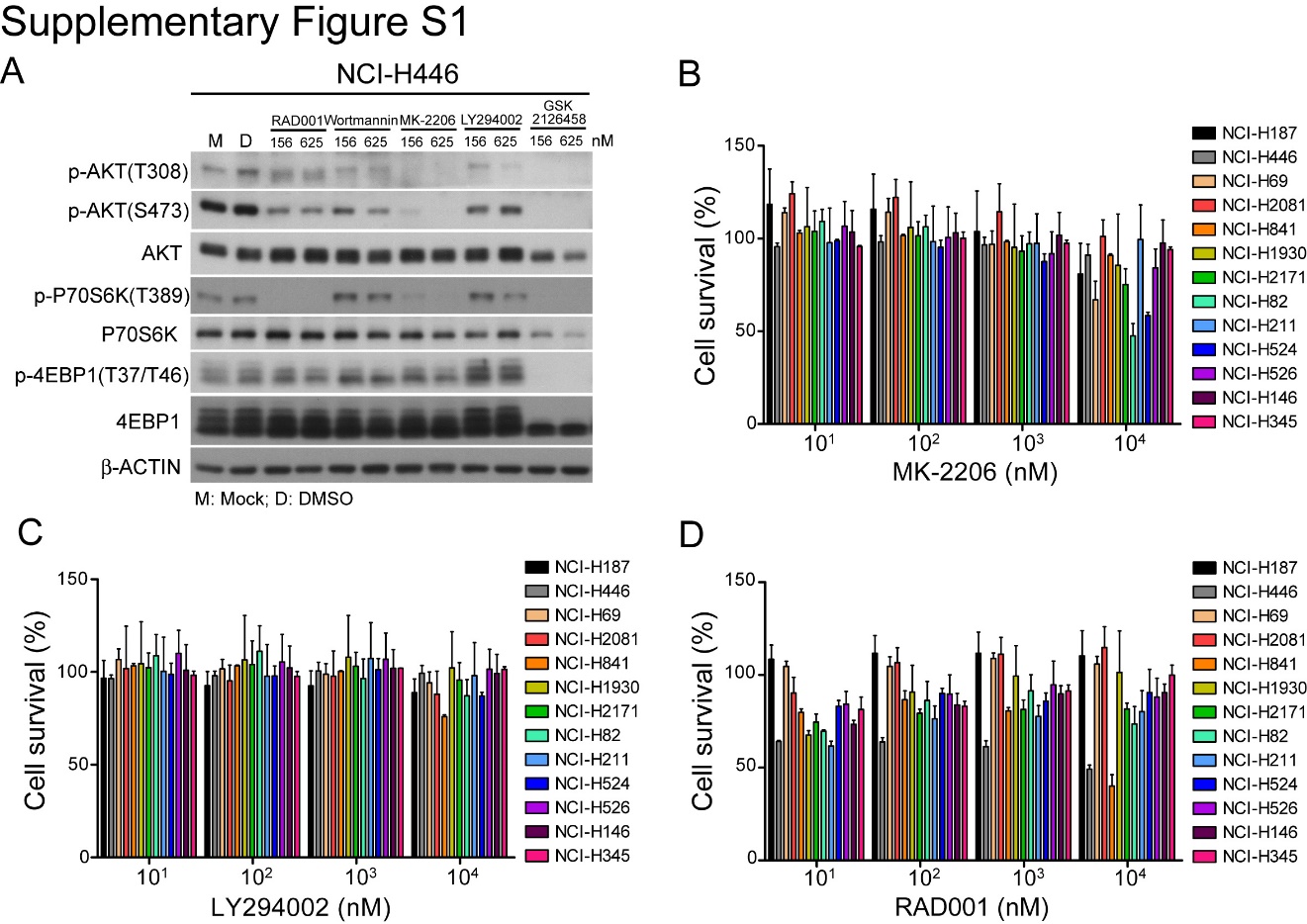
**

**Additional file 1: Figure S1.** Cell survival of SCLC cell lines treated using PI3K, AKT, or mTOR inhibitors. **A** Western blot analysis showing the expression of proteins involved in the PI3K/AKT/mTOR pathway in NCI-H446 cells treated using RAD001, Wortmannin, MK-2206, LY294002, and GSK2126458 at indicated concentrations for 24 h. **B-D** Percent survival of the thirteen SCLC cell lines in response to treatment using MK-2206 (**B**), LY294002 (**C**), and RAD001 (**D**).

**
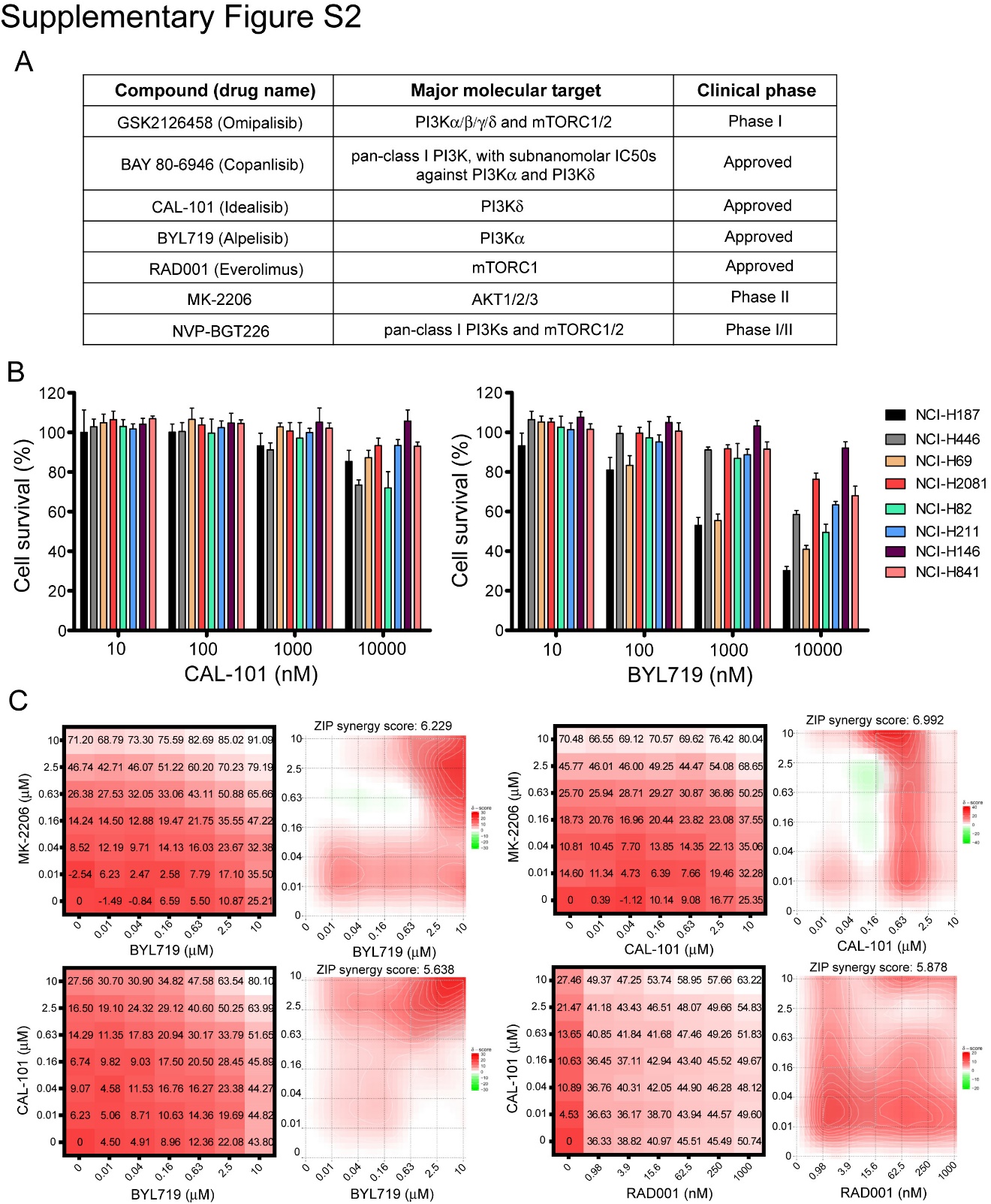
**

**Additional file 1: Figure S2.** Cell survival of SCLC cell lines treated using isoform-selective PI3K inhibitors. **A** Compound name and major molecular target of the PI3K inhibitors. **B** Percent survival of SCLC cell lines in response to treatment using CAL-101 or BYL719. **C** Dose matrix of drug combinations between any two of the following compounds: MK-2206, BYL719, RAD001, and CAL-101. Percent growth inhibition at each dose assessed after drug treatment for 72 h was shown on left. Synergy distribution and synergy scores based on a ZIP model were shown on right.

**
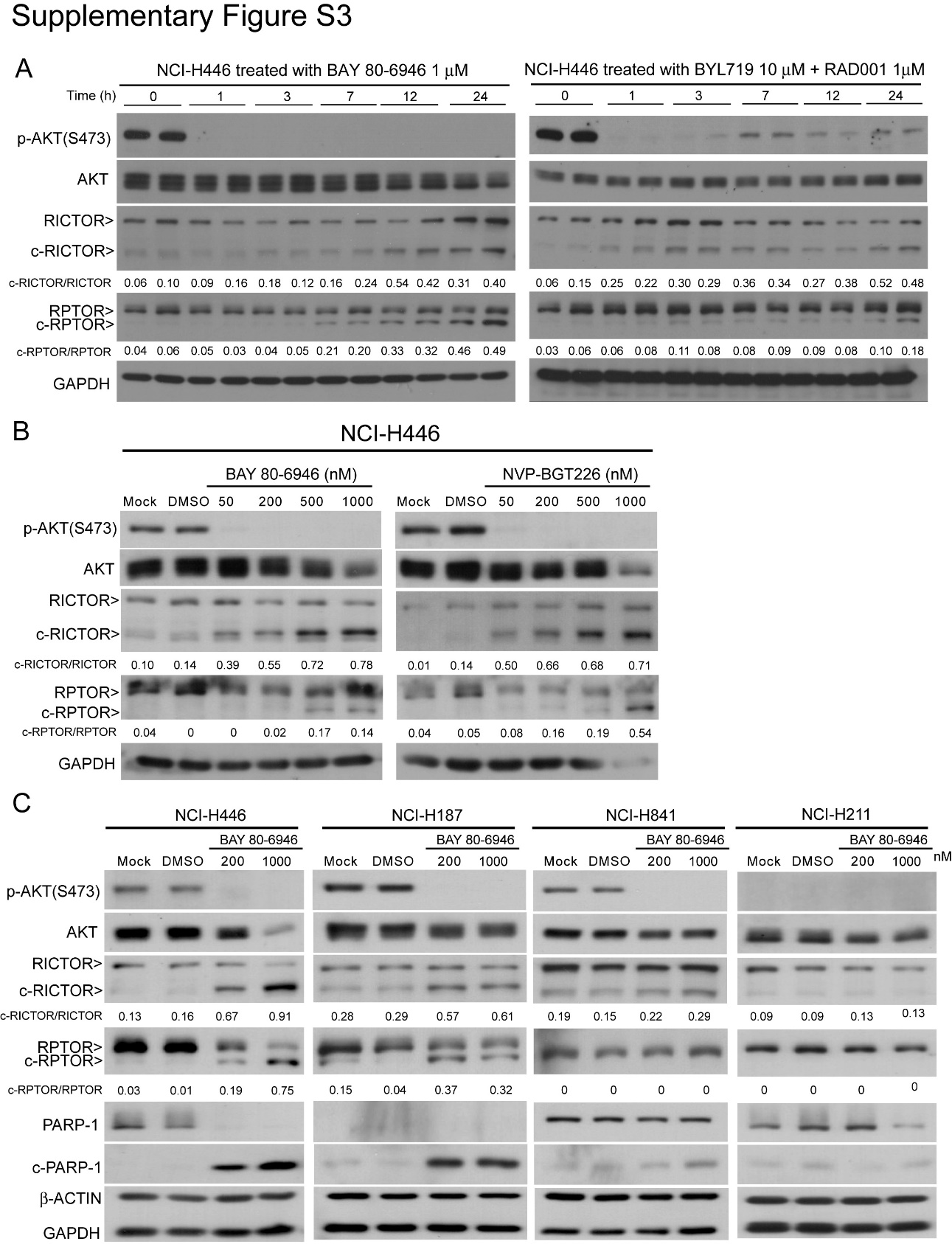
**

**Additional file 1: Figure S3.** Proteolytic cleavages of RICTOR and RPTOR in NCI-H446 cells treated using PI3K/mTOR inhibitors. **A** Western blot analysis showing the time-dependent cleavages of RICTOR and RPTOR in NCI-H446 cells treated using BAY 80-6946 (1 μM) or BYL719 (10 μM) and RAD001 (1 μM) in combination. **B** Western blot analysis showing the expression of AKT, p-AKT(S473), RICTOR, and RPTOR in NCI-H446 cells treated using BAY 80-6946 or NVP-BGT226 at indicated concentrations for 24 h. **C** Western blot analysis showing the expression of AKT, p-AKT(S473), RICTOR, and RPTOR in high p-AKT (NCI-H446 and NCI-H187), medium p-AKT (NCI-H841), and low p-AKT (NCI-H211) cells treated using BAY 80-6946 at indicated concentrations for 24 h. The expression levels of c-RICTOR and c-RPTOR were quantified and listed below the profiles in (**A**), (**B**) and (**C**).

**
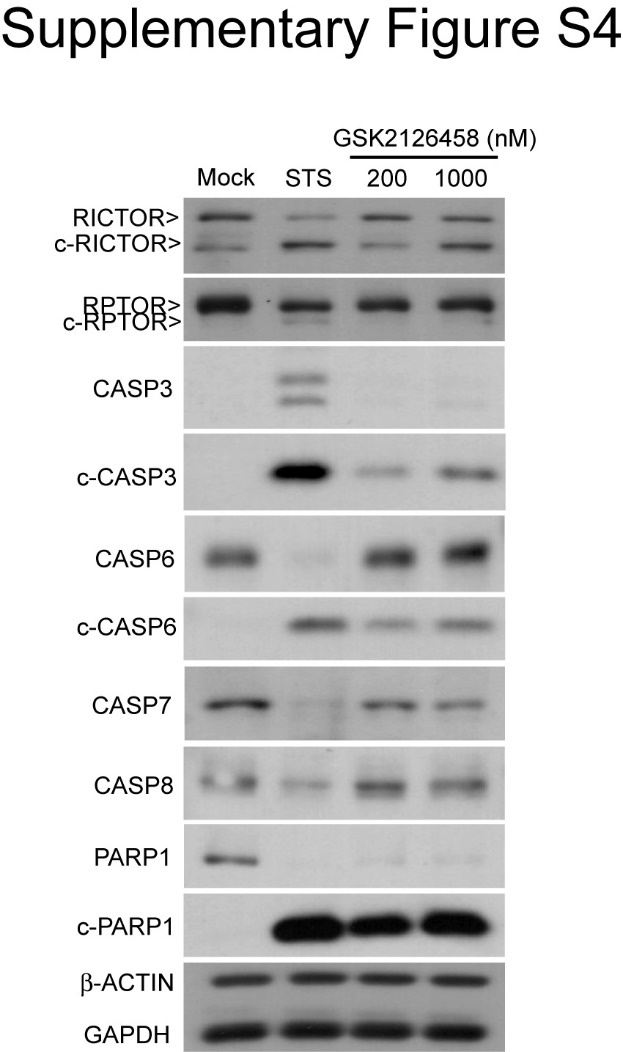
**

**Additional file 1: Figure S4.** Activation of caspases in high p-AKT cells via dual PI3K/mTOR inhibition. Western blot analysis showing the cleavage of RICTOR and RPTOR and the expression of caspases in NCI-H446 cells treated using Staurosporine (STS, 2 μM) or GSK2126458 (200 and 1,000 nM) for 7 h.
